# Herpes simplex encephalitis in Iceland 1987–2011

**DOI:** 10.1186/2193-1801-3-524

**Published:** 2014-09-13

**Authors:** Heiður Mist Dagsdóttir, Bryndís Sigurðardóttir, Magnús Gottfreðsson, Már Kristjánsson, Arthur Löve, Guðrún Erna Baldvinsdóttir, Sigurður Guðmundsson

**Affiliations:** Faculty of Medicine, School of Health Sciences, University of Iceland, Reykjavik, Iceland; Department of Infectious Diseases, Landspítali University Hospital, Reykjavik, Iceland; Department of Virology, Landspítali University Hospital, Reykjavik, Iceland

**Keywords:** Viral encephalitis, Herpes simplex virus, Herpes simplex encephalitis, Neurological sequelae

## Abstract

Herpes simplex encephalitis (HSE) is a serious disease with 10-20% mortality and high rate of neuropsychiatric sequelae. This study is a long-term, nationwide study in a single country, Iceland. Clinical data were obtained from patient records and from DNA PCR and antibody assays of CSF. Diagnosis of HSE was classified as definite, possible or rejected based on symptoms, as well as virological, laboratory and brain imaging criteria. A total of 30 definite cases of HSE were identified during the 25 year period 1987-2011 corresponding to incidence of 4.3 cases/106 inhabitants/year. Males were 57% of all patients, median age 50 years (range, 0-85). Fever (97%), cognitive deficits (79%), impaired consciousness (79% with GCS < 13), headache (55%) and seizures (55%) were the most common symptoms. Brain lesions were found in 24 patients (80%) by MRI or CT. All patients received intravenous acyclovir for a mean duration of 20 days. Three patients (10%) died within one year and 21/28 pts (75%) had a Karnofsky performance score of <70% with memory loss (59%), dysphasia (44%), frontal symptoms (44%) and seizures (30%) as the most frequent sequelae. Mean delay from onset of symptoms to treatment was 6 days; this was associated with adverse outcome. In conclusion, the incidence of `HSE is higher than recently reported in a national registry study from Sweden. Despite advances in rapid diagnosis and availability of treatment of HSE, approximately three of every four patients die or are left with serious neurological impairment.

## Introduction

Herpes simplex virus (HSV) causes various infections in humans, including encephalitis. HSV is the most common cause of non-epidemic encephalitis worldwide (Granerod et al. 2010; Banatvala 2011). Herpes simplex encephalitis (HSE) is a rare disease. The majority of cases (>90%) are due to HSV type 1 (HSV-1) and HSV type 2 (HSV-2) is the main cause of neonatal HSV infection (Aurelius et al. 1993). In a recent Swedish nationwide register-based study the incidence of confirmed HSE was 2.2 cases per million population per year (Hjalmarsson et al. 2007). The mortality of patients with HSE is 70% in the absence of treatment and most survivors have severe neurological impairment (Whitley et al. 1977). In the past 3 decades, two major improvements have improved mortality and morbidity of patients with HSE. In the mid-1980s two large randomized trials showed that treatment with intravenous acyclovir (ACV) reduced the mortality rate and decreased morbidity (Whitley et al. 1986; Skoldenberg et al. 1984). The establishment of polymerase chain reaction (PCR)–based diagnosis of HSV in cerebrospinal fluid (CSF) has facilitated faster and more reliable diagnosis of the disease, replacing the previous gold standard of brain biopsy (Aurelius et al. 1991; Lakeman and Whitley 1995; Scoular et al. 2002; Cinque et al. 1996). Furthermore, diagnostic accuracy has improved markedly with advances in neuroradiological imaging, in particular magnetic resonance imaging (MRI) (Domingues et al. 1998). Despite these advances the mortality is 15–20% and prevalence of neurological sequelae may range from 20–50 (Whitley et al. 1986; Raschilas et al. 2002).

Within the past decade several studies have improved our understanding of the epidemiology and etiology of encephalitis and the relative incidence of HSE as a cause of the acute encephalitis syndrome (Glaser et al. 2003; Barbadoro et al. 2012). In addition, the Swedish register-based study mentioned above was the first published nationwide study on HSE proper with regard to incidence, mortality and patients’ outcome. The objective of our study was to describe the epidemiology of HSE in a nationwide cohort in Iceland over the past 25 years, and to analyze clinical parameters such as main symptoms, diagnostic studies, treatment and outcomes directly from patient records.

## Methods

### Setting

Iceland has a national health care system, with comprehensive hospital registries. The population in 1987 was 245.962 and 319.014 in 2011 (Statistics Iceland). There are two referral hospitals in the country, the Landspitali University Hospital in Reykjavik and the Akureyri Hospital in Northern Iceland.

All cases of serious illnesses suggestive of encephalitis are referred to these two hospitals for diagnosis and management.

### Patients

All potential cases of HSE in Iceland from January 1987 through December 2011 were reviewed. Patients were identified by discharge diagnoses (according to International Classification of Diseases, Ninth and Tenth Revision, ICD-9 and ICD-10; ICD9 054.3, 323.4; ICD10 B00.4, G05.1, A86). In addition, all cases diagnosed with a positive HSV DNA PCR or by detection of intrathecal HSV specific antibody production in CSF were reviewed. Analysis of intrathecal antibody index (IAI) was performed by calculating the ratio of HSV antibodies in CSF relative to HSV antibodies in serum, as well as measuring antibodies for measles virus for comparison to rule out breach in the blood–brain barrier (Jacobi et al. 2007). The PCR assay for HSV was introduced in 1994 but until January 1998 it did not allow for typing into types 1 and 2. Separation into types was also not possible with the IAI assay performed during the whole study period.

By this approach 91 cases were identified. Their charts were reviewed retrospectively and clinical parameters registered, including symptoms, lesions identified on computed tomography (CT), magnetic resonance imaging (MRI) and electroencephalography (EEG), treatment and sequelae. State of consciousness was determined by the Glasgow coma scale (GCS) (Teasdale and Jennett 1974) and outcome was assessed using the Karnofsky performance scale measuring functional impairment (KPS) (Karnofsky et al. 1948). In both instances the patients were retrospectively scored based on information in the patient record at discharge, most often from the rehabilitation unit or from last documentation of contact in the record.

The study was approved by the National Bioethics Committee (ref. no. VSNb2011110005/03.7) and the Icelandic Data Protection Agency (ref. no. 201111138ÞS/-).

### Criteria

The diagnosis of HSE was established by using criteria developed by the authors to assess whether a patient had definite or possible diagnosis. Those who did not fulfill these criteria were excluded from the study. The following criteria were used: **Major criteria** were defined as 1) positive PCR for HSV or elevated IAI in CSF, 2) positive brain biopsy and 3) positive imaging study (CT or MRI) suspicious for HSE. **Minor criteria** were defined as 1) seizures, 2) cognitive deficits, 3) fever, 4) elevated white or red blood cells in CSF and 5) positive EEG findings.

Definite diagnosis of HSE required the patient to have two major criteria, one major and three minor criteria or all of the five minor criteria and no alternative diagnoses. Possible diagnosis of HSE was defined as one major and two minor criteria or 3–4 minor criteria and no likely differential diagnoses.

**Figure 1:**
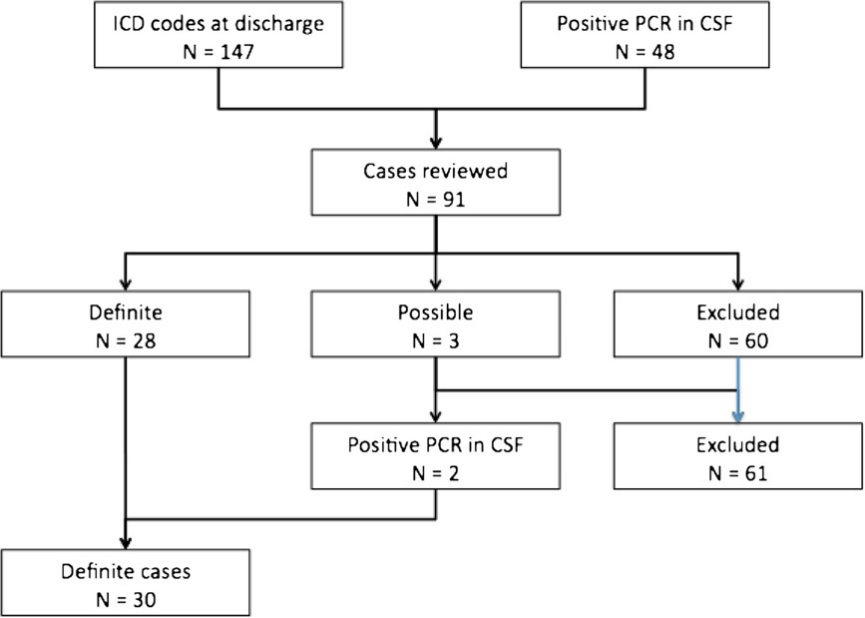
**Flow chart showing the study’s population.**

These methods led to finding of 28 definite cases and three possible cases from 1987 through 2011. The three possible cases all occurred before 1993. Their CSF samples were retrieved from storage and PCR for HSV-1 performed, yielding two positives. Thus, the final cohort of patients consisted of 30 definite cases of HSE (Figure 1).

### Statistics

The incidence of HSE was calculated based on population data from Statistics Iceland (Statistics Iceland). The statistical software packages SAS (SAS Institute Inc., Cary, North Carolina, USA, 2010) and R (Prolink Corporation, Naples, Florida, USA, 2009) were used to calculate confidence intervals, perform logistic regression, chi square test and Fisher test as appropriate. Two-tailed p values of <0.05 were considered to indicate statistical significance.

## Results

### Incidence

**Figure 2:**
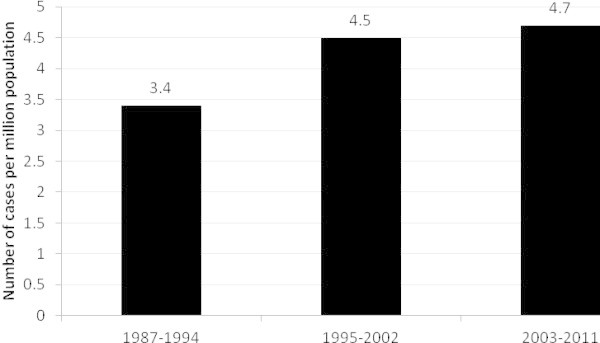
**Changes in incidence in the period 1987–2011, p > 0.05.**

Out of 91 patient records reviewed, a total of 30 patients were identified as having a definite diagnosis of HSE during the 25-year period 1987- 2011 (Figure 1). This corresponds to an incidence of 4.3 cases of HSE per million population per year (95% CI, 2.9–6.1 cases per million population per year). Figure 2 shows incidence over three 8 and 9 year periods. There was neither significant variation in incidence between those periods (p = 0.78) nor trend over time (p = 0.51). No significant difference in incidence between seasons was noted (p > 0.05).

### Virological diagnosis

HSV PCR assay was performed on the CSF of 25 of the patients in the cohort, 22 had positive PCR and 3 negative. During the study period a total of 48 patients had a positive PCR of CSF. The other 26 patients with positive HSV PCR who did not fulfill the criteria for HSE all had HSV meningitis (Figure 1). CSF PCR was not done on 5 patients who presented before the introduction of PCR. Thus, 8 patients in the cohort either had a negative PCR or it was not performed. Five of those 8 patients had a positive IAI, but 3 patients were diagnosed based on other criteria, two had one major and three minor criteria, and one patient had five minor criteria.

### Age and gender

**Figure 3:**
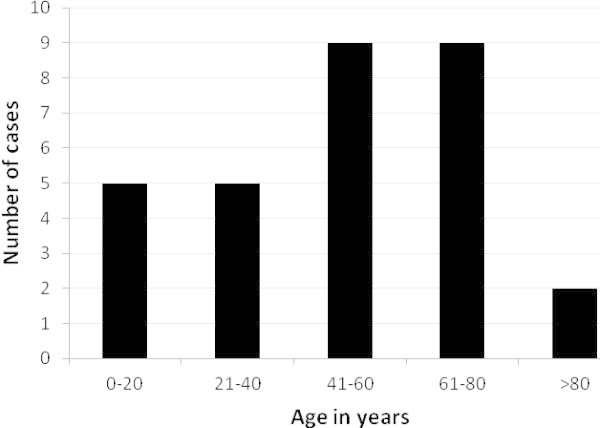
**Age distribution of patients.**

Of the 30 patients, 17 (57%) were males. Patients’ ages ranged from 0 to 85 years with a mean of 47 years (SD, 24 years) and median of 50 years (interquartile range (IQR), 34–68 years). The median age was 46 years in female patients (IQR, 22–72 years) and 51 years in male patients (IQR, 44–66 years). Age-specific incidence for the entre cohort is shown in Figure 3.

### Clinical features

Information about clinical features of all the patients was obtained. Fever was the most common feature (97%) and the average temperature was 38.6°C (STD, 0.8°C). Seventeen patients (57%) had seizures and 23 patients (79%) had one or more symptoms of cognitive impairment, of which confusion was the most common (76%). Twenty-four patients had maximum or medium alteration of consciousness over the course of the illness, i.e. a score of less than 12 on the GCS, and of those, 13 patients (43%) had GCS score of less than 8.

### Cerebrospinal fluid

All but one patient (97%) had an elevated white blood cell count (>3 × 10^6^/L) in the CSF with a median of 74 × 10^6^/L (IQR, 17–213 × 10^6^/L). Of those, 12 patients (40%) had more than 100 × 10^6^/L WBC. All but five patients had an increased amount of red blood cells (>1 × 10^6^/L) in CSF and five patients (17%) had more than 100 × 10^6^/L RBC in the CSF. Seventeen (57%) patients had increased level of protein (>600 mg/L) in the CSF with an average amount of 841 mg/L (STD, 521 mg/L).

### Imaging

Thirteen patients (43%) had only CT scans whilst the remaining 17 patients (57%) had an MRI. Diagnostic imaging (MRI and CT) showed lesions in 24 cases (80%), of which all but one patient (97%) had lesions in the temporal lobe (Table 1).

**Table 1:** **Number and ratio of patients who had CT or MRI along with topography of lesions**

	Total pts	Ratio
**Total number of patients receiving imaging study**	30*	**100%**
**Normal CT or MRI**	6**	**20%**
**Abnormal CT or MRI**	24***	**80%**
Temporal lobe****	23	96%
Other than temporal lobe*****	1	4%
Lesion in one hemisphere	19	79%
Lesions in both hemispheres	5	21%

*17 patients had MRI and 13 patients only CT.

**1 patient with MRI and 5 patients with only CT.

***16 patients with MRI and 8 patients with only CT.

****Lesions in temporal lobe with or without lesions in other sites of the brain.

*****One patient had no lesions in the temporal lobes, only lesions confined to the occipital lobe and corpus callosum.

### Treatment

**Figure 4:**
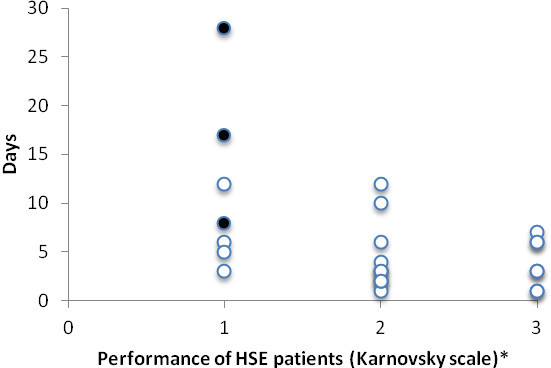
**Distribution of delay (days) in treatment with intravenous ACV from the onset of symptoms (y-axis) compared to prognosis after HSE (x-axis).** The OR of being in Karnofsky performance scale (KPS) category 2 and 3 vs. KPS category 1 for each day of delay is 0.76 (95% CI 0.55–0.94p = 0.04). Black dots indicate patients who died within a year from admission. *1 = 0–40% performance. 2 = 50–70% performance. 3 = 80–100% performance.

All patients received intravenous ACV for an average of 20 days (SD, 7 days). Information about morbidity and impairment after HSE was available for 28 patients (93%). Average time delay from the onset of symptoms until treatment with intravenous ACV was initiated was six days (SD, 6 days) and median four days (IQR, 3–8 days). This information was available for 28 patients. The association between duration of symptoms until treatment initiation and functional status after the illness is shown in Figure 4. The OR of being in KPS category 2 and 3 vs. KPS category 1 for each day of delay is 0.76 (95% CI 0.55–0.94, p = 0.04). The mean delay from admission to initiation of treatment was 2 days (SD, 5 days) and median 1 day (IQR, 0–2 days). Four patients received steroids.

### Outcome

Three (10%) patients died within one year (95% CI, 2–27%), all female. Seven of a total of 28 patients (25%) for which information on performance outcome was available had 80–100% ability to carry out activities of daily living based on the KPS scale, 14 patients (50%) had 50–70% ability and seven patients (25%, including the four who died) had a KPS of 0–40%. Thus, 75% of the patients either died or were left with serious sequelae. Of patients diagnosed during the first half (1987–1999) and the second half (2000–2010), respectively, 83% and 71%, respectively, were left with a KPS score of 70% or less (p = NS). Information on specific sequelae was available for 27 patients, of those a total of 23 (85%) had some sequelae, one or more, as shown in Table 2. Steroid therapy (4 patients) did not influence outcome.

**Table 2:** **Morbidity after HSE**

Sequelae	Number	Ratio
Memory loss	16	59%
Dysphasia	12	44%
Frontal symptoms	12	44%
Seizures	8	30%
Deep venous thrombosis	1	4%

Number of patients is shown with each impairment and the ratio of total number of patients, (n = 27).

## Discussion

In this long-term, population-based study the yearly incidence of HSE was 4.1 cases/1.000.000 inhabitants. If the single neonatal HSV-2 case is omitted, the incidence of HSV-1 encephalitis was 4.1 cases/1.000.000 inhabitants. This is considerably higher than the incidence of 2.2 recently reported from Sweden for the years 1990–2001 (Hjalmarsson et al. 2007). This discordance could reflect a true difference between the two countries, but the method of case acquisition is also a potential explanation. The Swedish study was based on ICD coded diagnoses for HSE in the Swedish National Inpatient Register that has covered all Swedish hospitals since 1987. Hospital-based inpatient registries are also available in Icelandic hospitals, but only 17 of a total of 29 patients (59%) with HSE in the current study would have been identified by the registry diagnoses for HSE alone. The remaining 12 cases were identified from other ICD codes, but mainly from registry of results of PCR testing and/or intrathecal antibody production in CSF at the only virology laboratory in the country. Cases of HSE in the Swedish study that were not typed as HSV-1 or 2 were not included in the analysis, but if they had been included the yearly incidence would have been 2.5/1.000.000 inhabitants (Hjalmarsson et al. 2007).

In the current study, we made an attempt to apply a set of rigorous criteria for inclusion, taking into account the fact that in clinical practice the diagnostic approaches to this disease may be different, especially given the long study period. The diagnostic criteria developed by the authors have a corollary in criteria employed for other diseases, e.g. the modified Duke criteria for bacterial endocarditis,(Li et al. 2000). Thus, patients without firm virological diagnosis were included in the study cohort according to the diagnostic criteria, akin to patients with culture negative endocarditis.

If the inclusion criteria used by Hjalmarsson et al. are applied to the current study population, the annual incidence in Iceland for 1999–2011 is 2.8/1.000.000, which is within the confidence limits of the Swedish study. A recent hospital-based registry from Italy 1999–2005 showed that an annual HSE incidence of 3.9/million inhabitants), very similar to the results in the current study (Barbadoro et al. 2012). Thus, although it is well known that the incidence of certain infectious diseases differ significantly by geography, it is very likely that the true incidence of HSE in both countries is very similar, but the methodology in the current study was less restrictive.

Three of 24 patients had negative PCR results. One of them presented outside the country, where facilities for PCR were not available. Three weeks after onset of symptoms he was transferred to Iceland. At that time the PCR was negative, as it may be 10–14 days after onset of symptoms (Puchhammer-Stockl et al. 2001). The diagnosis was made based on intrathecal antibody index (IAI). The other two patients were included based on other clinical or laboratory criteria. It is well known that patients with HSE may have an initially negative PCR and therefore the test should be repeated in patients in whom the clinical suspicion is high (Weil et al. 2002).

IAI was determined in seven patients, with a significant increase demonstrated for five. The remaining two patients both had a positive PCR for HSV. Thus, the IAI may have been tested too early in the course of the illness or it may be less sensitive than PCR.

Determinations of HSV types by PCR were begun at our facilities in 1998, and prior to that, types were not determined by antibody assays. Nevertheless, we included non-typed HSV in the study (Aurelius et al. 1993). This approach is justified given the fact that beyond the neonatal period, HSE is almost entirely caused by HSV-1 as demonstrated by Raschilas et al. (2002).

Fever was an almost universal symptom in our patients, and cognitive dysfunction, convulsions and headache were furthermore very common, as reported by others (Raschilas et al. 2002; Whitley et al. 1982). Interestingly, in the prospective study by Granerod and colleagues, fever was present on admission in only 50% of the HSE patients (Granerod et al. 2010). Forty-one percent of patients in our study were found to have markedly impaired consciousness (GCS score of <8) sometime in the course of illness as compared with only 10% of the patients in the study by Raschilas et al. (2002). However, the score in that study was determined on admission. We report on the other hand the lowest score during the course of the illness. In addition, the score in our study was determined in many cases retrospectively based on descriptions in the patient record, which certainly could bias our findings.

All but five patients had red blood cells in the CSF, as reported by others (Raschilas et al. 2002; McGrath et al. 1997). However, only 55% of our patients had increased protein level in the CSF, lower than reported by others (Raschilas et al. 2002; Whitley et al. 1982). One-fifth of our patients had normal imaging studies of the brain, but not everyone underwent MRI, which is a more sensitive diagnostic method than CT scan, especially for detecting early changes (Schroth et al. 1987). The lesions that were found were mostly in the temporal lobe, unilateral in 78% and bilateral in 22%, in concordance with previous reports (Raschilas et al. 2002). In one patient the lesion was in the occipital lobe resulting in significant visual loss.

All our patients received acyclovir, for mean duration of 20 days. The mean delay from onset of symptoms and initiation of treatment was 6 days and the delay from hospital admission until treatment was 2 days, similar to the delays reported by Raschilas et al. (2002). Furthermore, a significant association between duration of treatment delay and adverse outcome was demonstrated as reported previously by others (Raschilas et al. 2002; McGrath et al. 1997).

Three patients died within one year from admission, or 10% which is concordant with other studies (Granerod et al. 2010; Hjalmarsson et al. 2007; Raschilas et al. 2002). Approximately 70% of the surviving patients were left with disability following their illness, including memory loss, frontal symptoms, dysphasia and seizure disorder, in accordance with previous reports (Granerod et al. 2010; Hjalmarsson et al. 2007; McGrath et al. 1997; Stahl et al. 2012). Of interest is the recent study by Mailles et al. where only 42% of patients with HSE had a long term (3 year) favorable outcome, in contrast to patients with encephalitis due to other infectious causes, 68% of which had a favorable long-term outcome (Mailles et al. 2012).

The main strength of this study is that case finding was not limited to national or hospital registers only, but also on a comprehensive review of virological data. A case definition was made based on predetermined criteria, which take clinical, imaging and laboratory parameters into account. It was not uncommon for patients with a positive PCR for HSV to have the diagnosis of encephalitis at discharge although the symptoms and clinical course indicated meningitis. The extent of this miscoding in register-based studies is unclear, but it can have a major effect on the results. In this study, records from all possible cases were reviewed, in lieu of the criteria, thus strengthening the case finding process. In three cases it was possible to obtain old CSF samples for PCR testing to verify the diagnosis. Moreover, the study encompasses a whole nation and the duration of the study period is 25 years. Thus, it involves all verified cases in a whole country over a quarter of a century.

HSE is a relatively rare disease. The main limitations are the small population and the retrospective nature of the study from preexisting case records, which are subject to bias. Furthermore, retrospective assessment of GCS and KPS is imprecise as initial data collection and documentation may have been incomplete. Marked progress in the diagnosis of HSE took place during the study period, particularly the introduction of PCR technology. It is possible that we missed cases occurring prior to that time, but the incidence was not significantly lower in the early years of our study and therefore this seems unlikely.

In summary, in spite of the advent of rapid diagnostic technology and progress in antiviral treatment, HSE remains a very serious disease with a mortality rate of 10% and approximately three-fourths of the survivors suffer from serious sequelae. Therefore, further investigations into its risk factors, epidemiology, pathobiology, as well as improvements in diagnosis and more effective treatments are of utmost importance.

## References

[CR1] Aurelius E, Johansson B, Skoldenberg B, Staland A, Forsgren M (1991). Rapid diagnosis of herpes simplex encephalitis by nested polymerase chain reaction assay of cerebrospinal fluid. Lancet.

[CR2] Aurelius E, Johansson B, Skoldenberg B, Forsgren M (1993). Encephalitis in immunocompetent patients due to herpes simplex virus type 1 or 2 as determined by type-specific polymerase chain reaction and antibody assays of cerebrospinal fluid. J Med Virol.

[CR3] Banatvala JE (2011). Herpes simplex encephalitis. Lancet Infect Dis.

[CR4] Barbadoro P, Marigliano A, Ricciardi A, D’Errico MM, Prospero E (2012). Trend of hospital utilization for encephalitis. Epidemiol Infect.

[CR5] Cinque P, Vago L, Dahl H, Brytting M, Terreni MR, Fornara C, Racca S, Castagna A, Monforte AD, Wahren B, Lazzarin A, Linde A (1996). Polymerase chain reaction on cerebrospinal fluid for diagnosis of virus-associated opportunistic diseases of the central nervous system in HIV-infected patients. AIDS.

[CR6] Domingues RB, Fink MC, Tsanaclis AM, de Castro CC, Cerri GG, Mayo MS, Lakeman FD (1998). Diagnosis of herpes simplex encephalitis by magnetic resonance imaging and polymerase chain reaction assay of cerebrospinal fluid. J Neurol Sci.

[CR7] Glaser CA, Gilliam S, Schnurr D, Forghani B, Honarmand S, Khetsuriani N, Fischer M, Cossen CK, Anderson LJ, California Encephalitis Project, 1998-2000 (2003). In search of encephalitis etiologies: diagnostic challenges in the California Encephalitis Project, 1998-2000. Clin Infect Dis.

[CR8] Granerod J, Ambrose HE, Davies NW, Clewley JP, Walsh AL, Morgan D, Cunningham R, Zuckerman M, Mutton KJ, Solomon T, Ward KN, Lunn MPT, Irani SR, Vincent A, Brown DWG, Crowcroft NS, on behalf of the UK Health Protection Agency (HPA) Aetiology of Encephalitis Study Group (2010). Causes of encephalitis and differences in their clinical presentations in England: a multicentre, populationbased prospective study. Lancet Infect Dis.

[CR9] Hjalmarsson A, Blomqvist P, Skoldenberg B (2007). Herpes simplex encephalitis in Sweden, 1990–2001: incidence, morbidity, and mortality. Clin Infect Dis.

[CR10] Jacobi C, Lange P, Reiber H (2007). Quantitation of intrathecal antibodies in cerebrospinal fluid of subacute sclerosing panencephalitis, herpes simplex encephalitis and multiple sclerosis: discrimination between microorganism-driven and polyspecific immune response. J Neuroimmunol.

[CR11] Karnofsky DA, Abelmann WH, Craver LF, Burchenal JH (1948). The use of nitrogen mustards in the palliative treatment of cancer. Cancer.

[CR12] Lakeman FD, Whitley RJ (1995). Diagnosis of herpes simplex encephalitis: application of polymerase chain reaction to cerebrospinal fluid from brain-biopsied patients and correlation with disease. National Institute of Allergy and Infectious Diseases Collaborative Antiviral Study Group. J Infect Dis.

[CR13] Li JS, Sexton DJ, Mick N, Nettles R, Fowler VGJ, Ryan T (2000). Proposed modifications to the Duke criteria for the diagnosis of infective endocarditis. Clin Infect Dis.

[CR14] Mailles A, De Broucker T, Costanzo P, Martinez-Almoyna L, Vaillant V, Stahl JP (2012). Long-term outcome of patients presenting with acute infectious encephalitis of various causes in France. Clin Infect Dis.

[CR15] McGrath N, Anderson NE, Croxson MC, Powell KF (1997). Herpes simplex encephalitis treated with acyclovir: diagnosis and long term outcome. J Neurol Neurosurg Psychiatry.

[CR16] Puchhammer-Stockl E, Presterl E, Croy C, Aberle S, Popow-Kraupp T, Kundi M, Hofmann H, Wenninger U, Godl I (2001). Screening for possible failure of herpes simplex virus PCR in cerebrospinal fluid for the diagnosis of herpes simplex encephalitis. J Med Virol.

[CR17] Raschilas F, Wolff M, Delatour F, Chaffaut C, De Broucker T, Chevret S, Lebon P, Canton P, Rozenberg F (2002). Outcome of and prognostic factors for herpes simplex encephalitis in adult patients: results of a multicenter study. Clin Infect Dis.

[CR18] Schroth G, Kretzschmar K, Gawehn J, Voigt K (1987). Advantage of magnetic resonance imaging in the diagnosis of cerebral infections. Neuroradiology.

[CR19] Scoular A, Gillespie G, Carman WF (2002). Polymerase chain reaction for diagnosis of genital herpes in a genitourinary medicine clinic. Sex Transm Infect.

[CR20] Sköldenberg B, Alestig K, Burman L, Forkman A, Lövgren K, Norrby R, Stiernstedt G, Forsgren M, Bergström T, Dahlqvist E, Frydén A, Norlin K, Olding-Stenkvist I, Uhnoo I, De Vahl K (1984). Acyclovir versus vidarabine in herpes simplex encephalitis. Randomised multicentre study in consecutive Swedish patients. Lancet.

[CR21] Stahl JP, Mailles A, De Broucker T (2012). Herpes simplex encephalitis and management of acyclovir in encephalitis patients in France. Epidemiol Infect.

[CR22] Statistics IcelandAvailable from: http://www.statice.is/Statistics/Population. Accessed July 16, 2013

[CR23] Teasdale G, Jennett B (1974). Assessment of coma and impaired consciousness. A practical scale. Lancet.

[CR24] Weil AA, Glaser CA, Amad Z, Forghani B (2002). Patients with suspected herpes simplex encephalitis: rethinking an initial negative polymerase chain reaction result. Clin Infect Dis.

[CR25] Whitley RJ, Soong SJ, Dolin R, Galasso GJ, Chien LT, Alford CA (1977). Adenine adenine arabinoside therapy of biopsy-proved herpes simplex encephalitis — National Institute of Allergy and Infectious Diseases collaborative antiviral study. N Engl J Med.

[CR26] Whitley RJ, Soong SJ, Linneman C, Liu C, Pazin G, Alford CA (1982). Herpes simplex encephalitis. Clinical assessment. JAMA.

[CR27] Whitley RJ, Alford CA, Hirsch MS, Schooley RT, Luby JP, Aoki FY, Hanley D, Nahmias AJ, Soong SJ (1986). Vidarabine versus acyclovir therapy in herpes simplex encephalitis. N Engl J Med.

